# Stem Cells: The Pursuit of Genomic Stability

**DOI:** 10.3390/ijms151120948

**Published:** 2014-11-14

**Authors:** Saranya P. Wyles, Emma B. Brandt, Timothy J. Nelson

**Affiliations:** 1 Center for Regenerative Medicine, Mayo Clinic, Rochester, MN 55901, USA; E-Mails: wyles.saranya@mayo.edu (S.P.W.); brandt.emma@mayo.edu (E.B.B.); 2 Center for Clinical and Translational Sciences, Mayo Clinic, Rochester, MN 55901, USA; 3 Department of Molecular Pharmacology and Experimental Therapeutics, Mayo Clinic, Rochester, MN 55901, USA; 4 Division of General Internal Medicine, Mayo Clinic, Rochester, MN 55901, USA

**Keywords:** pluripotent stem cells, multipotent stem cells, DNA damage response, reprogramming efficiency, apoptotic susceptibility

## Abstract

Stem cells harbor significant potential for regenerative medicine as well as basic and clinical translational research. Prior to harnessing their reparative nature for degenerative diseases, concerns regarding their genetic integrity and mutation acquisition need to be addressed. Here we review pluripotent and multipotent stem cell response to DNA damage including differences in DNA repair kinetics, specific repair pathways (homologous recombination *vs.* non-homologous end joining), and apoptotic sensitivity. We also describe DNA damage and repair strategies during reprogramming and discuss potential genotoxic agents that can reduce the inherent risk for teratoma formation and mutation accumulation. Ensuring genomic stability in stem cell lines is required to achieve the quality control standards for safe clinical application.

## 1. Introduction

Stem cells have the capacity for self-renewal and differentiation into endoderm, mesoderm, and ectoderm tissues. The promise for human disease treatment using pluripotent stem cells, including embryonic stem cells (ESCs) and induced pluripotent stem cells (iPSCs), also carries the threat of genomic instability. Previous reviews have addressed this concern for genomic integrity and reported several factors that contribute to differences in genomic and epigenomic stabilities of stem cells, including derivation source (embryonic *vs*. somatic cells), derivation methods (direct isolation *vs*. reprogramming), and culture conditions [1]. Additionally, mechanisms to protect stem cell genomes from endogenous and exogenous genotoxic stresses have been outlined [2]. Studies have found that DNA damage and repair mechanisms govern the reprogramming efficiency of somatic cells back to pluripotency. Indeed, several DNA repair pathways, including homologous recombination (HR) and non-homologous end joining (NHEJ), contribute to the maintenance of genomic stability and stem cell quality. DNA damage response pathways that regulate genomic integrity have been evaluated in multipotent and pluripotent stem cell populations, and allow them to differentiate into multiple cell types without propagating DNA repair errors. Utilization of stem cells for regenerative medicine imparts the pre-requisite of fully comprehending the stress response in pluripotent and multipotent stem cell lines.

Stem cells hold the prospective capability to restore function to diseased and aged tissues, yet further research is needed to elucidate the response of these cells to genotoxic agents. Endogenous adult and/or exogenous transplanted stem cells could be subject to DNA damaging irradiation depending on treatment protocols and disease recurrence. The main concerns about stem cells and their differentiated derivatives are their ability to restore physiological functionality and their ability to resist neoplastic transformation in response to endogenous genotoxic stress. In the promising era of stem cell research and therapy, ensuring genomic stability of stem cells remains one of the highest priorities prior to clinical translation. In this review, we provide a brief summarization of pluripotent and multipotent stem cell responses to DNA damage including differences in DNA repair kinetics, specific repair pathways (HR *vs.* NHEJ), and apoptotic sensitivity. We also describe current studies evaluating DNA damage and repair strategies during reprogramming in addition to highlighting known and novel factors that regulate reprogramming efficiency. Furthermore, we discuss recent reports that utilize genotoxic agents for iPSC therapeutic development.

## 2. DNA Damage and Repair Status during Reprogramming

**Figure 1:**
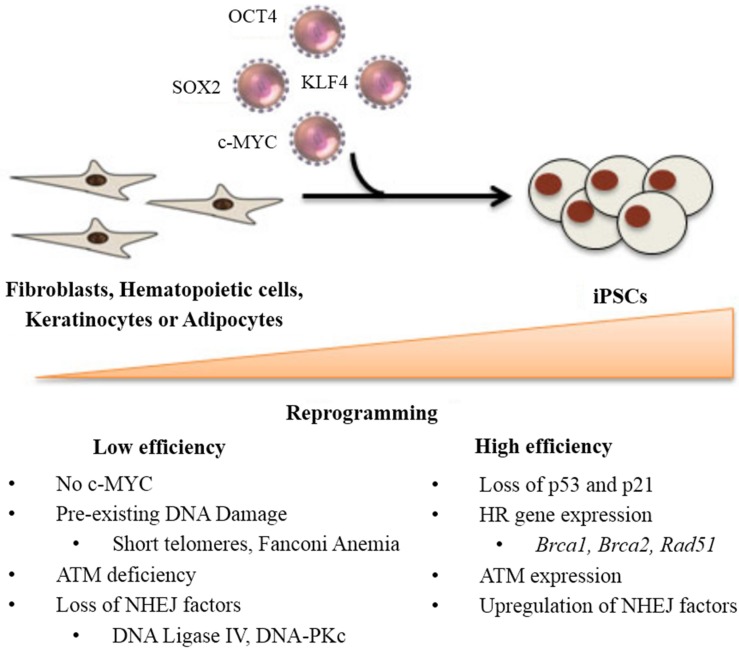
DNA damage factors that govern reprogramming efficiency from the somatic cell state to the pluripotent state are summarized. High efficiency is achieved with downregulation of apoptotic factors including p53 and upregulation of DNA repair genes (homologous recombination (HR) and non-homologous end joining (NHEJ)). Pre-existing DNA damage in combination with low DNA repair capacity leads to low efficiency.

iPSCs were initially derived using retroviral vectors encoding the factors OCT4, SOX2, KLF4, and c-MYC that successfully reprogrammed somatic cells back into a pluripotent state [3,4]. Multiple cell types, including fibroblasts, hematopoietic lineages [5,6], keratinocytes [7], and adipocytes [8] have been reprogrammed to pluripotency. Despite the great potential of this technology, one of the continued hurdles for iPSC generation is its low efficiency of reprogramming (<1%) [9]. Studies have shown that reprogramming without c-MYC can achieve pluripotency, yet its efficiency is even lower [10]. To address this challenge, several investigators demonstrated that loss of p53 contributed to an increase in the efficiency of reprogramming [11,12]. Indeed, p53 is involved in DNA damage response and apoptosis [13]. It plays a crucial role in preventing the propagation of DNA-damaged cells [14]. Hong *et al*. [11] suggest that the suppressive effects of tumor suppressor genes, p53 and p21, on cell proliferation and survival contribute to direct effects on cellular reprogramming. Similarly, Marion *et al.* [12] show that p53 constitutes a main barrier to reprogramming, especially exacerbated in cells with pre-existing DNA damage, such as short telomeres. Suboptimal cells with DNA damage are eliminated by p53-dependent apoptotic response and prevented from becoming pluripotent stem cells [12]. In accordance, recent studies show that decreasing p53 protein levels increased generation of iPSCs using only OCT4 and SOX2 [15]. Hence, while permanent suppression of p53 could lower the quality of iPSCs and cause genomic instability, transient suppression by siRNA or similar methods could be useful in attaining higher efficiency of reprogramming (Figure 1) [11,16].

Further investigation of patient-specific samples deficient in DNA repair enzymes demonstrated that an intact DNA damage response is critical for iPSC reprogramming. For instance, ataxia telangiectasia mutated (*Atm*^−/−^) mice, which accumulate mutations and fail to respond to DNA double-strand breaks (DSB) [17], were studied for reprogramming potential. Indeed, ATM is a protein kinase that phosphorylates a number of target proteins including p53 [18]. Kinoshita *et al.* showed that *Atm*^−/−^ tail-tip fibroblasts (TTF) reprogrammed at 1/50th of the efficiency from wild-type mice TTFs, indicating that *Atm* does participate in the reprogramming process [19]. Additionally, *Atm*^−/−^ iPSCs were more sensitive to stress and accumulated severe chromosomal abnormalities after several passages, demonstrating its pertinent role in maintaining chromosomal stability [19]. Recently, ATM-deficient human pluripotent stem cells have been established and were predictably associated with extremely low reprogramming efficiency [20,21]. ATM-dependent phosphorylation directly regulates p53 activation and also affects a broad range of substrates such as DNA repair, apoptosis, G1/S, intra-S checkpoint and G2/M checkpoints [22]. Thus, ATM and p53 appear to have significant roles on the reprogramming of differentiated cells to pluripotency. Mechanisms behind the DNA repair switch that occurs upon reprogramming to pluripotency and how reprogramming can revert DNA repair functionalities are still unknown. Utilizing systems biology-based technologies to profile the reprogramming factors’ interactome could provide answers [23].

Decreased reprogramming efficiency has also been observed due to the loss of HR genes. To assess whether reprogramming is a trigger of DNA damage, recent studies investigated the role of the HR DNA repair pathway during reprogramming and subsequent expansion of iPSC clones in culture [24]. Gonzalez *et al.* [24] showed that HR genes, including *Brca1*, *Brca2*, and *Rad51*, are required for efficient reprogramming, using both integrative or non-integrative methods, and their loss decreased efficiency when using non-integrating vectors. In contrast, Soyombo *et al.* report that it was easier to reprogram mutant patient-specific BRCA1 fibroblasts than the fibroblasts from relatives without the mutation [25]. Further investigation is required to understand whether this difference is due to the HR gene mutation, homozygous *vs.* heterozygous, or to clonal variations in generating iPSC lines.

In addition to the HR pathway, the role of NHEJ in reprogramming of human somatic cells to iPSCs and in regulation of their differentiation has been investigated. Tilgner *et al*. studied this major DBS repair pathway using iPSC lines from patients with LIG4 syndrome due to DNA ligase IV mutations [26]. Indeed, DNA ligase IV is required for the end processing step of NHEJ [27]. This study demonstrated that reprogramming efficiency was significantly reduced when DNA ligase IV was mutated. Furthermore, the limited reprogramming capacity was associated with genomic instability and could be rescued by reintroduction of DNA ligase IV [26] or genetic complementation [28]. Interestingly, p53 downregulation did not have any effect on the reprogramming defect in mutated iPSC lines, leading to the possibility that cell death induced by impaired DNA repair in DNA ligase IV-lacking cells could be p53 independent [29]. It is possible that the stress of reprogramming leads to DNA DBS formation, yet the exact mechanism remains to be elucidated. Moreover, understanding the DNA damage response in iPSCs is complicated by the fact that the process of reprogramming could increase the DNA damage load [2].

Similar to DNA ligase IV, other factors in NHEJ such as DNA-dependent protein kinase catalytic subunit (DNA-PKc), also showed a four-fold to seven-fold decrease in reprogramming efficiency and reduced capacity for clonal expansion [30]. Accordingly, iPSC generation from Fanconi anemia patients with inactivating mutations that increase susceptibility to DNA damage displayed a ten-fold reduction in reprogramming efficiency that could be alleviated by correcting the gene mutation [31]. Taken together, these studies highlight the importance of DNA repair in successful iPSC generation.

Interestingly, these investigations were conducted with the use of lentiviral or retroviral reprogramming methods. The integration steps of these viruses require DNA DSB for successful viral replication [32]. Park *et al.* recently published an improved method for protein reprogramming that increased genomic integrity of mouse iPSC lines compared to retroviral and lentiviral strategies [33]. Additional non-integrating methods have been developed to circumvent issues related to insertional mutagenesis including recombinant proteins [34,35], mRNA [36,37], microRNA [38,39], and non-integrating viruses such as adenovirus [40] and Sendai virus [41]. Further studies using non-integrating reprogramming methods are needed to accurately assess the role of the DNA damage response in iPSC generation. It remains unknown whether these pathways are the result of the retroviral activity or if the reprogramming process is inherently stressful to genomic integrity. Two of the reprogramming factors, *Oct4* and *Sox2*, were seen to associate with the nucleotide-exchange-repair (NER) complex as co-activators on pluripotency-related genes [42]. This study provides some light into the molecular association between maintaining genomic integrity and sustaining the pluripotent state, but deserves further research. Comparing the process of oocyte-mediated reprogramming or somatic cell nuclear transfer (SCNT) may also provide some insight into how the early zygote maintains its genomic integrity. A recent study screened for factors that could decrease DNA damage response signals during oocyte-induced reprogramming and identified *Zscan4* as a factor that promotes genomic stability, telomere elongation, and improved reprogramming efficiency [43,44]. Indeed, *Zscan4* stabilized genomic DNA, resulting in p53 and p21 downregulation [43,45]. Hence, DNA damage response and repair strategies that promote efficiency of iPSC generation and maintain its genomic stability could allow us to improve the overall quality of iPSC lines for clinical and laboratory applications.

## 3. Stem Cell Response to DNA Damage

DNA damage response among various stem cell populations constitutes an important facet of stem cell safety and efficacy for regenerative purposes. In multipotent or adult stem cell populations, many studies have assessed DNA damage response to exogenous DNA damaging factors such as ionizing radiation (IR), X-rays, and chemotherapeutic agents. Similarly, in pluripotent stem cell populations, DNA damage response has been evaluated with pronounced differences in DNA repair capacities compared to multipotent stem cells. These differences include the kinetics of the DNA repair, the preferred correction pathways such as HR *vs.* NHEJ, and apoptotic sensitivity (Table 1).

**Table 1:** Summary of single-strand and double-strand break repair strategies and apoptosis sensitivity in pluripotent and multipotent stem cell populations compared to differentiated somatic cells.

Stem Cell Type	Single-Strand Breaks	Double-Strand Breaks		Apoptosis Sensitivity	References
		HR	NHEJ		
**Pluripotent**					
Human ESCs, iPSCs	++	+++	++	+++	[46,47,48,49,50,51,52,53,54,55,56,57,58]
Mouse ESCs, iPSCs	*	+++	−	+++	[59,60]
**Multipotent**					
Neural stem cells	*	+++	++	++	[57,61,62,63,64,65]
Mesenchymal stem cells	*	+++	+++	−	[63,66,67]
Hematopoietic stem cells	++	*	++	+++	[68,69,70]

ESCs, embryonic stem cells; iPSCs, induced pluripotent stem cells; (+++) indicates a substantial increase compared to differentiated somatic cells; (++) indicates a moderate increase compared to differentiated somatic cells; (−) indicates a decrease compared to differentiated somatic cells; and (*) indicates lack of published data.

### 3.1. Pluripotent Stem Cells

Pluripotent stem cells, including ESCs [71] and iPSCs, can differentiate into all three embryonic germ layers. Therefore their ability to maintain genomic stability, by stress defense and DNA repair, is highly regulated as genetic alterations can compromise competency for lineage commitment and development. To mitigate genomic instability caused by DNA damage, pluripotent stem cells employ two main strategies: (1) Hypersensitivity to DNA damaging agents leading to apoptosis; and (2) enhanced activity of DNA repair proteins and DNA DSB repair. This biological contradiction in stem cells is well established, however the reasoning remains speculative. It is possible that enhanced DNA repair capacity is merely a response to high growth rate and more endogenous DNA damage. Pluripotent stem cells display an abbreviated cell cycle with a short G phase and longer S phase. The need for pluripotent cells to protect their genome for future generation is accomplished both through this enhanced DNA repair and apoptotic sensitivity.

Following exposure to DNA damaging agents (H_2_O_2_, UV-C, IR, psoralen), human ESCs show more efficient DNA repair than differentiated fibroblasts and HeLa cells [46]. Maynard *et al*. used markers of DNA repair including reactive oxygen species 8-Oxoguanine (8-OXOG) levels, 8-Oxoguanine Glycosylase (OGG1) incision activity, and expression of DNA repair genes, to quantify the response to DNA damage [46]. Similarly, mouse ESCs demonstrate greater resistance to DNA damage from oxidative stress and IR than mouse fibroblasts [60]. Stress defense mechanisms such as telomerase activity and antioxidant genes are downregulated with lineage specification [58]. These findings validate the evolutionary theory that stress defense and DNA repair mechanisms are most active in germ line cells, including ESCs that can differentiate into germ line cells, however it would be energetically unfavorable in somatic cells.

Compared to somatic cell lines, human ESCs have an abbreviated cell cycle with a short G1 period suggesting that mechanisms regulating DNA damage response may differ from their differentiated counterparts. The G1 checkpoint functions to protect genomic integrity by preventing cells with damaged DNA from entering S-phase. Filion *et al*. expanded on this hypothesis and showed that human ESCs lack a G1 checkpoint in response to IR-induced DNA damage but block the G2 phase of the cell cycle [47]. In response to DNA damage, human ESCs initiated apoptosis and underwent cell death via Caspase-related mitochondrial apoptosis [47,72]. In contrast, Barta *et al.* showed that human ESCs irradiated in G1 phase undergo cell cycle arrest prior to DNA synthesis, demonstrating their ability to activate the G1/S checkpoint with damage to their genetic component [73]. Others have described human ESCs with a leaky G1/S checkpoint [74]. Further clarification of ESC cell cycle regulation could aid efforts to eliminate damaged cells and maintain an error-free stem cell population.

Pluripotent stem cells have also developed mechanisms to effectively repair DNA DSBs. Several groups have shown that HR mechanism governs repair in both mouse and human ESCs [49,51,57,59]. Increased expression of DNA repair genes at the transcriptional and protein level [52] is seen in pluripotent cells compared to differentiated cells. Few studies have examined upstream signaling of the DNA damage response in pluripotent cells. For example, Adams *et al*. suggest that pluripotent cells may have preferences for the DNA damage sensors like ataxia telangiectasia and Rad3-related protein (ATR) for high DNA repair capacity [49]. In addition to HR-mediated DSB repair, Adams *et al*. recently demonstrated NHEJ functionality in human ESCs [50]. Human iPSCs mimic human ESCs in their repair protein expression and NHEJ repair efficacy, indicating reprogramming of DNA damage response pathways [52]. Studies in human ESCs have also found that NHEJ repaired radiation-induced DSBs during the late G2 stage of the cell cycle [48]. Moreover, DSB repair, either by HR or NHEJ, is highly precise in human ESCs, compared to either the somatic human cells or mouse ESCs [53]. Alternatively, in mouse ESCs, HR is the predominant DSB repair pathway with minimal contribution from error prone NHEJ [59]. The opposite is true in differentiated cell lines. Additional pathways such as DNA mismatch repair (MMR) were reported to be robust in pluripotent stem cells with high sensitivity to DNA alkylation damage [75]. Comparatively, in cancer cells, both NHEJ and HR compete with each other to facilitate DSB repair, and it is still poorly understood what dictates which pathway is used [76].

Despite these active repair mechanisms, overwhelming amounts of DNA damage causes a shift within stem cells from survival through repair to death by apoptosis of damaged cells [77]. In fact, some studies suggest that pluripotent stem cells control genome integrity by apoptosis rather than DNA repair. Desmarais *et al.* showed that direct measurements of single stranded DNA (ssDNA) fail to generate ssDNA regions necessary for HR repair initiation, committing to apoptosis instead [54]. Luo *et al*. found that pluripotent stem cells including human ESCs and iPSCs were more prone to apoptosis than differentiated progenitors despite enhanced repair rates [55]. Recently, Liu *et al*. showed that human ESCs undergo more rapid p53-dependent apoptosis after DNA damage than differentiated cells and attributed mitochondrial priming, defined as the mitochondrial readiness for apoptosis, to stem cell sensitivity upon DNA damage [78]. While the majority of human ESCs succumb to apoptosis, a subpopulation continues to proliferate with damaged DNA and point mutations, leading to genetically aberrant human ESCs [56]. It is therefore crucial to understand DNA repair capacities in pluripotent stem cells and ensure genomic stability prior to its utilization in regenerative applications.

### 3.2. Multipotent Stem Cells

Adult stem cells are tissue-resident, multipotent stem cells with limited differentiation potential. Damage to genomic DNA in multipotent stem cells elicits activation of the DNA damage response and initiates DNA repair, cell cycle arrest, and ultimately apoptosis or cellular senescence [79,80]. DSB repair kinetics varies among cell types [81] with recent findings supporting faster repair rates in NSCs compared to terminally differentiated astrocytes [61]. In accordance, NSCs were more resistant to IR-induced DNA DSBs than differentiated neurons [62]. Interestingly, NSCs that survived high doses of IR underwent cellular senescence, a stable permanent cell cycle arrest [82]. Similar reports have noted apoptosis, elevated metabolic activity, and radiation sensitivity post-irradiation [57,63,83]. Previous studies suggest that NSCs are highly prone to p53-dependent apoptosis following IR [64,84,85,86,87]. In contrast, Schneider *et al*. recently report that NSCs undergo a delayed and p53-independent apoptosis upon irradiation, which can be suppressed by caspase inhibition and Bcl2 overexpression [65,88]. Prior to this apoptotic response, the primary mode of DNA repair in NSCs is through HR and response efficacy is directly linked to cell cycle phase during which the DNA damage occurred [64]. Compared to NHEJ, HR is a more accurate method to repair DSB and has great importance for NSCs during brain development [89]. Understanding the ability for NSCs to resist mutation accumulation remains crucial in the development of therapies targeting neurodegenerative diseases.

Multipotent mesenchymal stem cells (MSCs) have also been characterized with higher resistance to DNA damage [66]. Indeed, MSCs are typically derived from bone marrow or adipose tissue and have the ability to differentiate into other cells of mesenchymal origin, including osteoblast, chondrocytes, and adipocytes [90]. Although DNA damaging agents including cisplatin and γ-irradiation are widely used in the treatment of hematogical malignancies, limited knowledge exists on their effects on MSCs [91,92]. Prendergast *et al.* show that direct DNA damage to human MSCs activates key repair pathways, including NHEJ, and cell cycle checkpoints [66]. Additionally, the type and extent of DNA damage could dictate whether or not human MSCs retain their ability for lineage specification [63]. In order to regulate human MSC differentiation after DNA damage, apoptosis and DNA repair have been proposed as the major safeguard mechanisms [67]. Given the growing role of MSCs in clinical trials for degenerative diseases and its endogenous contribution to regeneration, future studies to elucidate DNA damage response mechanisms would be beneficial.

In mammalian tissue with modest regenerative potential, it is known that endogenous stem cells endure an age-dependent decline in function [93,94]. At the molecular level, evidence supports the accumulation of DNA damage in tissue stem cells [95,96,97]. Hematopoietic stem cells (HSCs) isolated from the blood or bone marrow can differentiate into blood and immune cells. HSCs have been studied extensively yet molecular mechanisms that regulate its response to DNA damage remain largely unknown [98]. Wang *et al*. recently identified a differentiation checkpoint that controls HSC differentiation in response to DNA damage [68]. This G-CSF/Stat3/BATF-dependent cell cycle checkpoint induces lymphoid differentiation in response to DNA damage or telomere dysfunction.

Other methods to maintain HSC chromosomal integrity include NHEJ-mediated repair. Recently, thrombopoietin, a regulator of platelet production, was shown to promote DNA repair and increase NHEJ efficiency, suggesting that HSC environmental factors can contribute to DSB repair machinery [69]. Additionally, cell autonomous mechanisms also contribute to DNA damage response in the HSC population. Cho *et al.* utilized the ERCC1-XPF DNA repair endonuclease (*Ercc1^−/Δ^*) mice, which accumulate endogenous DNA damage, to show increased cell death, senescence markers, and reactive oxygen species in HSCs [70]. Adult stem cells are particularly susceptible to DNA lesions due to constant genotoxic stress from endogenous processes. Hence, characterizing cell autonomous and non-autonomous mechanisms to DNA repair would be valuable and especially relevant for patients that require bone marrow transplantation.

### 3.3. Summary of Single-Strand (ss-) and Double-Strand (ds-) Breaks Repair Strategies

Overall, the type of repair used by pluripotent and multipotent stem cells to restore DNA ss- and ds-breaks seem to be dependent on the cell cycle period or on the DNA damaging agent. Pluripotent stem cells are primed to undergo apoptosis but contain a robust and efficient DNA damage response compared to adult stem cells. Multipotent stem cells can also vary in apoptotic susceptibility depending on their niche environment and repair kinetics with MSCs being the most robust and HSCs being the most sensitive. DNA repair in pluripotent and multipotent stem cells utilized their differentiated progeny, including fibroblasts and neurons, to compare the differences in repair kinetics, accuracy, and expression levels of DNA repair genes. Overall, DNA repair is observed to be faster and more accurate in stem cell populations when compared to the DNA repair of their differentiated counterparts. There remain discrepancies in current literature in regards to preferred DNA repair pathways that need to be addressed.

## 4. Apoptotic Susceptibility in iPSC Therapeutic Development

In mammalian cells, DNA repair mechanisms preserve genomic integrity. However, deficient repair capacities can lead to genomic instability, which has been identified in human ESC lines [99,100,101,102] and iPSC lines [103,104]. In accordance, Hyka-Nouspikel *et al.* show that human ESCs lose genomic integrity and gain cancer-like phenotype following long periods of *in vitro* culture [56]. Reduced DNA damage response and cell cycle checkpoints lead to mutation accumulation in stem cell lines. Additionally, individual pluripotent cell lines demonstrate variability in innate DNA repair capacities [55]. Recent reports suggest that mouse iPSCs have higher sensitivity to radiation-induced DNA damage than mouse embryonic fibroblasts (MEFs), which could indicate lower DNA repair capacity and genomic instability [105]. It is possible that regulation of DNA damage repair response could reduce the risk of tumor formation that is inherent to pluripotency. Greater inspection of such lines is required prior to clinical use.

Distinguishing “good” *vs.* “bad” pluripotent stem cell lines on the scale of genomic stability can be achieved by measuring apoptotic susceptibility to genotoxic agents. Studies have shown that mouse ESCs are hypersensitive to DNA damaging agents and undergo apoptosis [106]. This high apoptotic response of ESCs to DNA damage could contribute to the reduction of mutational load in their subsequent progenitor population [107]. Similarly, human ESCs activate p53-dependent and -independent apoptotic machinery in response to DNA DSB [88,108]. DNA damage response and apoptosis induction has recently been recapitulated in human iPSCs, providing a natural mechanism to segregate pluripotent stem cells from their differentiated progeny [12,109]. While experimental evidence supports stem cell-based repair for degenerative diseases as well as heart disease [110,111,112], clinical translation is hindered by the inherent risk of dysregulated cell growth known as tumorigenicity [113]. To eliminate pluripotent stem cell-derived teratoma formation, previous studies have used iPSC-specific anti-apoptotic factors, such as survivin or Bcl10, with small molecule inhibitors (quercetin or YM155) and selectively induced apoptotic cell death of undifferentiated stem cells [114]. Similarly, inhibition of stearoyl-coA desaturase (SCD1), a key enzyme in oleic acid biosynthesis that induces ER stress and generates reactive oxygen species, eliminated undifferentiated iPSC derivatives [115]. Studies have also proposed the targeting of undifferentiated mouse ESCs and iPSCs using lentivirus-mediated thymidine kinase expression, which converts the prodrug ganciclovir into a toxic metabolite, leading to reduced teratoma formation [116]. Additionally, cytotoxic antibodies such as monoclonal antibody mAb 84 [117], which binds to podocalyxin-like protein-1 (PODXL) [118], have induced toxicity in undifferentiated pluripotent stem cells.

**Figure 2:**
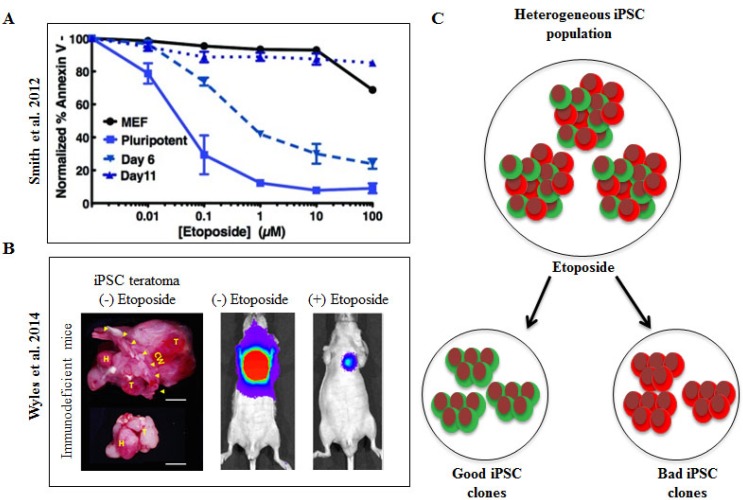
Utilization of genotoxic drug, etoposide, for iPSC therapeutic development. (**A**) Smith *et al.* demonstrated that etoposide can distinguish iPSC *vs.* differentiating cell populations, showing increased apoptotic susceptibility of iPSC clones compared to its differentiated day 6 and day 11 counterparts as well as mouse embryonic fibroblasts (MEFs) [119]; reprinted with permission from Stem Cells Translational Medicine, 2012, Volume 1, Issue 10, pp. 709–718, published by AlphaMed Press, Durham, NC, USA; (**B**) Wyles *et al.* recently demonstrated the risk of iPSC teratoma formation in the heart and provided evidence for lowering tumorigenicity with etoposide pre-treatment, as shown with their *in vivo* bioluminescence imaging in immunodeficient mice following acute myocardial infarction [121]; reprinted with permission from Stem Cells and Development, 2014, Volume 23, Issue 19, pp. 2274–2282, published by Mary Ann Liebert, Inc., New Rochelle, NY, USA. These studies suggest that it is possible to leverage apoptosis sensitivity and utilize etoposide as an adjunct therapy for iPSC-based cardiac repair; and (**C**) Based on this premise, etoposide could serve as a potential candidate to stratify the heterogeneous iPSC population into genetically stable “good” and unstable “bad” clones.

Pharmacological methods to purge pluripotent stem cells also offer a potential strategy to eliminate teratoma formation. Smith *et al.* utilized a DNA-damaging agent, etoposide, to purge pluripotent stem cells by means of apoptotic hypersensitivity following tissue-specific differentiation [119]. To address the concern of etoposide-induced genotoxic effects or permanent damage to the genome of tissue-specific progeny that could lead to secondary cancers, Smith *et al*. analyzed untreated and purged-surviving cells to differentiate into a cardiac lineage and demonstrated that both populations equally yield cardiomyocytes without causing genomic instability or loss of daughter cell differentiation potential [119]. Despite this observation, the anti-cancer agent, etoposide, has been reported to be associated with secondary leukemia in the clinical setting [120]. Recently, this pharmacological purging strategy was tested in the setting of iPSC-based myocardial therapy following acute myocardial infarction [121]. Wyles *et al*. [121] found that pre-treatment with genotoxic etoposide significantly lowered the threat of teratogenicity by purging the contaminating pluripotent cells. Indeed, DNA topoisomerase II inhibitor, etoposide, could be a potential candidate to discriminate “good” *vs.* “bad” stem cell clones in addition to being adjuvant therapy that reduces teratoma formation based on apoptosis sensitivity (Figure 2).

## 5. Conclusions

All stem cells are not created equally. Pluripotent and multipotent stem cell cultures are phenotypically heterogeneous and vary in their DNA damage response and apoptosis induction capacity. To overcome genomic aberrations and clonal heterogeneity, apoptotic susceptibility of stem cells, specifically ESCs and iPSCs, could be leveraged with genotoxic agents such as etoposide. Additionally, stratification of normal *vs.* mutation-prone stem cell clones will be essential to guarantee genomic stability. Current regenerative strategies require an optimized high-throughput validation to ensure the safety of stem cell-based therapeutics and to reach clinical reality.
